# Stress test: translational research during COVID‐19 pandemic

**DOI:** 10.1002/jcsm.12640

**Published:** 2020-11-18

**Authors:** Matthias Pumberger, Friederike Schömig, Henryk Haffer, Julia Mehl, Georg N. Duda

**Affiliations:** ^1^ Center for Musculoskeletal Surgery Charité—Universitätsmedizin Berlin Berlin Germany; ^2^ Julius Wolff Institute Charité—Universitätsmedizin Berlin Berlin Germany; ^3^ Berlin Institute of Health Center for Regenerative Therapies Charité—Universitätsmedizin Berlin Berlin Germany; ^4^ Berlin‐Brandenburg School for Regenerative Therapies Charité—Universitätsmedizin Berlin Berlin Germany

## Introduction

As part of the measures taken by the government to best contain the spread of SARS‐CoV‐2 and to protect staff members, German universities initiated, like most universities globally, a shutdown with only emergency operations remaining in place. This affected all research institutions, but specifically research in medicine, where non‐SARS‐CoV‐2‐associated research was banned from the lab infrastructure. On 17 March 2020, all research activities were suspended by the Berlin Senate Chancellery for Science and Research, unless there were valid reasons for continuation.^1^ Over the past weeks, several prestigious journals pointed out the importance of non‐COVID‐19 research during shutdown worldwide.^2^, ^3^, ^4^, ^5^, ^6^, ^7^ However, to our knowledge, no studies have been published regarding the pandemic's effect on personnel at different levels of their career in research institutions.

Since Germany has not been hit as heavily as expected and scientific and economical structures were in good shape prior to the pandemic, we were wondering how scientists unexpectedly confronted with the pandemic's effects by home office and lab shutdown would react. We initiated a questionnaire‐based survey across four research institutions in Germany to evaluate challenges scientists are facing at their stages of carrier. We chose to do so in an economically and politically stable country like Germany to focus on the effect introduced by the pandemic and to avoid any cross‐interactions with other social processes that could affect the evaluation. We aimed at obtaining objective data regarding personnel restructuring and financial changes and also the psychological distress caused by the pandemic.

## Study design, participants, survey, and statistics

In this cross‐sectional study, we conducted an online‐based anonymous voluntary survey within three research institutions of the largest German university hospital from 10 to 18 of May. These included the Institute for Medical Immunology (IMI), the Julius Wolff Institute (JWI), and the Berlin‐Brandenburg Center for Regenerative Therapies (BCRT), reaching a total of 230 employees. The study protocol was reviewed and approved by the institutional ethics committee (EA2/105/20).

We designed a questionnaire in German and English language, containing a total of 43 items. The first four questions addressed the participants' personal and professional profile using multiple‐choice questions on their age, sex, professional qualification, and professional experience. The following 39 questions were designed to evaluate the pandemic's impact and contributed to one of the following five indices: employer, professional impairment, personal impairment, society, and future. Questions regarding the different indices appeared mixed throughout the survey. A combination of a five‐degree or four‐degree Likert scales was used in most questions. Two questions were asked for estimation in per cent. The questionnaire was developed by the first and senior authors in internal consensus meetings and was revised and approved by external co‐authors. We used a bipolar 5‐point Likert scale with responses ranging from ‘totally disagree’ (corresponding to 1 on the scale), ‘rather disagree’ (corresponding to 2 on the scale), ‘neutral’ (corresponding to 3 on the scale), ‘rather agree’ (corresponding to 4 on the scale) to ‘totally agree’ (corresponding to 5 on the scale) and a bipolar 4‐point Likert scale with responses ranging from ‘never’ (corresponding to 1 on the scale), ‘on a few days’ (corresponding to 2 on the scale), ‘daily’ (corresponding to 3 on the scale) to ‘several times a day/night’ (corresponding to 4 on the scale). Data analysis was performed using Prism 5 (GraphPad Software, San Diego, CA, USA) and Microsoft Excel 2019 (Microsoft, Redmond, WA, USA). We performed descriptive analysis calculating the means and standard deviations where applicable.

## Results and discussion

A total of 163 employees participated (60.4%); 6.8% of participants were under 25 years old, 50.3% were between 25 and 34 years old, 29.2% were 35 to 44 years old, 10.6% were 45 to 54 years old, and 3.1% were over 55 years old; 66.5% were female and 33.5% male; and 38.5% indicated a master's degree to be their highest level of qualification, 26.1% were postdocs, 16.7% had a PhD, 9.9% were professors, and 8.7% had a bachelor's degree; 40.4% of participants had less than 3 years, 36.0% had 3 to 10 years, and 23.6% over 10 years of experience at their current institution. Overall results of participants regarding domains employer, professional impairment, personal impairment, society, and future are depicted in *Table* 1.

**Table 1:** Mean value of agreement (Likert scale 1–5, 1 indicating disagreement and 5 full agreement) depicted by participants different academic qualification regarding domains employer, professional impairment, personal impairment, society, and future

	Employer	Professional impairment	Personal impairment	Society	Future
Bachelor	3.7 ± 0.4	3.0 ± 0.5	3.2 ± 0.7	3.9 ± 0.2	3.2 ± 0.7
Master	3.9 ± 0.4	3.0 ± 0.6	3.2 ± 0.6	4.1 ± 0.2	2.9 ± 0.8
PhD	3.9 ± 0.4	3.0 ± 0.4	3.1 ± 0.7	4.1 ± 0.4	2.8 ± 1.0
Postdoc	3.9 ± 0.6	3.1 ± 0.5	3.2 ± 0.7	4.1 ± 0.3	2.8 ± 0.9
Professor	3.6 ± 0.8	3.2 ± 0.4	3.1 ± 0.9	4.4 ± 0.2	3.0 ± 1.0
Overall	3.8	3.1	3.1	4.1	2.9

The COVID‐19 pandemic has developed to one of the most drastic global challenges not only for societies in all kind of nations but also especially for their healthcare systems. While many reports have focused on frontline healthcare workers directly involved in the treatment of COVID‐19 patients, the challenges to scientists in research institutions have remained unknown so far. We on purpose conducted this cross‐sectional survey to identify the current constraints and demands of scientists in research institutions to provide insight into their current and future interpretation of the situation. It should allow conclusions for research institutions in the current pandemic setting as well as in future crises. We aimed at evaluating the personal strains experienced by researchers in this current dynamic situation in order to further analyse the need for psychological preventive strategies. We did so prior to the lifting of embargos and national constrains to get a reliable measure of the effects scientists that were confronted with a yet unsolved pandemic setting experienced.

Overall, despite a significant number of changes reported by the scientists from their work place, our study shows high satisfaction of researchers with the measures taken by their institutions to contain SARS‐CoV‐2 and to protect their employees. At this point of time and in the special German setting, staff shortages or forced leaves were rare and with a reported mean of 10.1%, and reduction of working hours was low, which explains the participants' positivity towards their institutions. This is consistent with the participants' agreement with the measures taken by the government.

In contrast to this rather positive impression and even though most participants did not report that they expect financial difficulties or financial threats due to the pandemic, 61.4% of participants were concerned about the further funding of their working position, showing a substantial uncertainty about the course of the pandemic and future changes in their specific—scientific focused—work environment. At the same time, expected reduction of income was low, and the majority of participants did not feel that the pandemic was a threat to their overall existence.

In their personal lives, concern about the pandemic was reported diversely, with almost equal numbers of participants reporting concern and no concern. The majority of participants felt restricted. However, overall self‐reported resilience was high, and fear and sleep disorders were reported at lower frequencies. It is, nevertheless, important to keep in mind that—so far—the number of infections and associated deaths was low in Germany, which may explain the low reported levels of fear and anxiety of employees in research institutions. We on purpose analysed scientists in medical research to allow a comparison to those working on the frontline in medical treatment. Here, even low numbers of anxious participants need to be noticed in order to readily implement support systems for those in need.

Interestingly, even though half of participants assumed that research activities would return to normal in the second half of 2020, 76.5% of them were concerned about not reaching planned goals and milestones and 42.6% reported fear regarding their future scientific career. This again shows very strong evidence that even under best possible starting conditions (German situation) and an otherwise rather stable setting (regarding current contract situations compared to many other employment conditions across the nation), participating researchers were not too concerned about the current state of the pandemic professionally but were concerned on the future course since the pandemic poses a relevant threat to their scientific achievement possibilities.

Our study shows that the measures taken in order to contain the SARS‐CoV‐2 pandemic led to massive changes not only in hospitals but also in associated research institutions. While short‐time work or discharge of employees was reported at low frequencies, home office was a concept applied by the majority of participants, assumedly leading to a reduction of onsite experiments. Therefore, not only the reported delay of achieving doctorates or professorships but also possibly more severely the delay of new scientific discoveries needs to be discussed. Additionally, most participants were concerned about presenting or publishing their scientific work, also showing that the current focus on COVID‐19 may reduce the scientific communication of non‐COVID‐19‐related research.

Some limitations of our study need to be mentioned. We focused on purpose on German research institutions in medicine. This makes a direct transfer of our results to other countries challenging, specifically if the initial economic conditions for scientists were different prior to start of the pandemic. A distortion of our results due to non‐response bias needs to be considered. Due to the dynamic course of the pandemic, we did not perform prior pilot testing of the survey. Also, this survey can only be seen as a first impression since the pandemic still is in its early stages in Germany and follow‐up surveys will give a more in‐depth analysis of the pandemic's impact.

Nevertheless, we show that despite the many measures restricting the work of research institutions in Germany and despite the financial and personal hardships faced by their employees, researchers agree with these changes and are satisfied with the government's and their institution's way of handling this crisis but see substantial challenges to come for their future career development possibilities. It can be expected that specifically the latter is even more dominant for scientists around the globe in those countries and institutions, in which the measures taken in Germany were not in place or had been installed in the course of the pandemic.

## Conflict of interest

None declared.
